# Gut microecological regulation on bronchiolitis and asthma in children: A review

**DOI:** 10.1111/crj.13622

**Published:** 2023-04-27

**Authors:** Sichen Xue, Rukkaiya Abdullahi, Naisheng Wu, Jishan Zheng, Miaoshang Su, Manhuan Xu

**Affiliations:** ^1^ Department of Pediatrics The First Affiliated Hospital of Ningbo University Ningbo Zhejiang China; ^2^ Department of Pediatric Respiratory Medicine The Second Affiliated Hospital and Yuying Children's Hospital of Wenzhou Medical University Wenzhou Zhejiang China; ^3^ Department of Pediatrics The Ningbo Women and Children's Hospital Ningbo China; ^4^ College of Laboratory Medicine and Life Science Wenzhou Medical University Wenzhou Zhejiang China

**Keywords:** asthma, bronchiolitis, children, gut microbiota, gut–lung axis

## Abstract

**Introduction:**

Asthma and bronchiolitis in children are considered common clinical problems associated with gut microbiota. However, the exact relationship between gut microbiota and the above‐mentioned diseases remains unclear. Here, we discussed recent advances in understanding the potential mechanism underlying immune regulation of gut microbiota on asthma and bronchiolitis in children as well as the role of the gut–lung axis.

**Methods:**

We retrieved and assessed all relevant original articles related to gut microbiota, airway inflammation‐induced wheezing in children, and gut–lung axis studies from databases that have been published so far, including PubMed/MEDLINE, Scopus, Google Scholar, China National Knowledge Infrastructure (CNKI) and the Wanfang Database.

**Results:**

The infant period is critical for the development of gut microbiota, which can be influenced by gestational age, delivery mode, antibiotic exposure and feeding mode. The gut microbiota in children with asthma and bronchiolitis is significantly distinct from those in healthy subjects. Gut microbiota dysbiosis is implicated in asthma and bronchiolitis in children. The presence of intestinal disturbances in lung diseases highlights the importance of the gut–lung axis.

**Conclusion:**

Gut microbiota dysbiosis potentially increases the risk of asthma and bronchiolitis in children. Moreover, a deeper understanding of the gut–lung axis with regard to the gut microbiota of children with respiratory diseases could contribute to clinical practice for pulmonary diseases.

## Graphical abstract

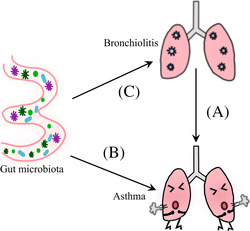

**Abbreviations**
APRIL: a proliferation‐inducing ligand
BAFF: B cell activation factor
BALF: bronchoalveolar lavage fluid
COPD: chronic obstructive pulmonary disease
C‐sections: caesarean sections
DCs: dendritic cells
Fut2: fucosyltransferase 2
GLA: gut–lung axis
GlcNAc: *N*‐acetylglucosamine
Gpr43: G protein‐coupled receptor 43
HMOs: human milk oligosaccharides
IBD: inflammatory bowel disease
IBS: irritable bowel syndrome
ICOS: inducible T‐cell co‐stimulator
IFNAR: IFN‐1 receptor
ILC3: 3 innate lymphoid cells
IL‐22: interleukin‐22
MLNs: mesenteric lymph nodes
MPP: *Mycoplasma pneumoniae* pneumonia
PRRs: pattern recognition receptors
RegIII: regenerated islet‐derived protein 3
RSV: respiratory syncytial virus
SAA: serum amyloid A
SCFAs: short‐chain fatty acids
SFB: segmental filamentous bacteria
Th1 cells: type 1 T helper cells
TLRs: toll‐like receptors

## 1 INTRODUCTION

Wheezing illness manifested by a continuous whistling sound during breath, suggests that some parts of the respiratory airways are narrow or obstructed, which is often led by airway inflammation, airway hyperresponsiveness, bronchospasm and airway remodelling. Among them, airway inflammation‐induced wheezing in children is common, including bronchial asthma, bronchiolitis and *Mycoplasma pneumoniae* pneumonia (MPP), with the first two being the most frequent causes.^1^, ^2^ Mucosal immunity participates in the formation of the first defending line in the respiratory system. The development and maturation of the early pulmonary immune system are determined by the host, environment and gut microbiota.^3^ The intestinal tract is the largest immune organ harbouring microbiota that is widely distributed on the intestinal surface. The gut microbiota not only communicates with the host to promote the maturation of the mucosal immune system but also maintains intestinal homeostasis.^4^ Accumulative evidence suggests that the establishment of the gut microbial community in early childhood may be important for the future health of children.^5^ Despite the regulatory role of gut microbiota in the immune system components, dysbiosis in the gut microbiota can elicit abnormal immune responses and release various inflammatory factors, causing or aggravating airway inflammatory diseases. This review analyzed and discussed the role of gut microbiota in children with asthma and bronchiolitis, providing a reference for the modulation of gut microbiota in children with respiratory diseases.

## 2 METHODS

### 2.1 Outline of gut microbiota

The intestine is often referred to as the ‘second brain’ of our body, and the gastrointestinal tract harbours the majority of microbes that are essential for our health.^6^, ^7^, ^8^ The gut microbiota colonized on the surface of the intestinal mucosa is interdependent and symbiotic, playing a critical part in various physiological processes and promoting liver metabolism, tissue development and the regulation of the immune system.^9^, ^10^ The main phylum of mammalian gut microbiota includes Firmicutes, Bacteroidetes, Proteobacteria and Actinobacteria.^11^ Previous research has revealed that microbial colonization in the mammalian intestine allows the change of the original homogeneous community into body‐site‐specific communities, which has an important influence on the development of the human immune system.^12^, ^13^ A study conducted by Bäckhed et al.^14^ has suggested that the establishment of an anaerobic environment, availability of nutrients and microorganism interaction cause the consistent migration of microorganisms in the succession of infant gut microbiota. Recently, Gude et al.^15^ used *Escherichia coli* to investigate the coexistence of bacteria driven by the combination of motility and spatial competition. In the study, they found that there was a growth‐migration balance in the gut microbiota. In this case, the fast‐growing groups intended to occupy the original space, whereas the fast‐moving ones were more likely to reside in the remote area. This observation may explain how the intestine promotes ecological diversity.

### 2.2 Establishment, development and influencing factors of gut microbiota

It was originally believed that the gut microbiota did not colonize in the relatively sterile environment of the uterus. However, Jiménez et al.^16^ showed that the colonization of gut microbiota may begin even before birth. A scenario for the ‘placental microorganism’ is still controversial, and the period of microbial colonization in the intestine has not been determined.^17^ Kuperman et al.^18^ stated that although the placental microbiota present in infants are extremely low in biomass, the foetal environment in the uterus should not be considered sterile. Moreover, Rackaityte et al.^19^ found limited bacteria in the meconium. As one of the dominant bacterial groups, *Micrococcus* can adapt to the foetal environment and suppress inflammatory responses. It takes about 1 year for newborns to colonize the maternal bacteria to establish and develop their own gut microbiota.^20^ The transition of gut microbiota from facultative aerobe to anaerobe can be driven by a variety of factors, including gestational age, delivery mode, antibiotic exposure and feeding mode.^21^ In the case of delivery mode, the gut microbiota of infants born vaginally is similar to the vaginal microbiota of mothers, whereas the infants delivered via caesarean sections (C‐sections) have gut microbiota resembling their mother's skin microbiota.^6^ Notably, the babies born by C‐sections often have fewer beneficial gut bacteria, such as *Bacteroides* and *Bifidobacterium*, but more pathogenic bacteria present in their gut.^22^ It is important to note that though the delivery mode can affect health throughout adulthood, as the immune system gradually strengthens, the influence of the composition of gut microbiota gradually decreases over the years of birth. This finding indicates that early gut microbiota is linked to the growth and maturation of the host immune system.^12^, ^22^ Meanwhile, feeding style is another vital factor affecting gut microbiota in early life. Generally, breastfed infants have higher rates of *Bifidobacterium* and *Lactobacillus* in gut microbiota than formula‐fed infants because of the compounds contained in breast milk, such as immune active molecules, vitamins, probiotics and oligosaccharides.^23^, ^24^, ^25^ Forbes et al.^25^ demonstrated that formula feeding in babies appears to stimulate changes in microbiota associated with obesity. More importantly, gestational age has been defined as the principal driving force for gut microbiota development. The gut microbiota in pre‐term infants is different from those in full‐term infants, as shown by reduced healthy bacteria (*Bifidobacterium* and *Lactobacillus*) and increased pathogenic ones (*Staphylococcus* and *Enterococcus*) in the gut of pre‐term infants.^26^, ^27^ Korpela et al.^28^ found that hospital environment and breastfeeding are capable of restoring the gut microbiota of pre‐term infant to normal. Besides, antibiotics can cause short‐term changes in gut microbiota composition, which were normally restored to normal within 2–3 weeks. The sensitivity of gut microbiota to different antibiotics leads to the alteration of immune system reactivity. Another study revealed that although antibiotics vary in effects on the species richness of gut microbiota, the consumption of all antibiotics enriches the gut resistome and multi‐drug resistant microbiota.^29^ Strikingly, the use of antibiotics can not only weaken or completely eliminate the protection of breast milk against infection and overweight in infants but also reduce the long‐term breastfeeding‐caused promotion of the protective microflora.^30^

### 2.3 Regulation of gut microbiota on immune homeostasis

Gut microbiota can communicate with the host on the intestinal mucosa surface to coordinate with each other and achieve mutually beneficial symbiosis. The intestinal mucosa is continuously exposed to foreign antigens and colonized microorganisms. And the intestinal mucus layer, epithelial cells, lamina propria and gut commensal bacteria constitute a barrier that protects the intestine by restricting close contact between bacteria and intestinal epithelial cells. The above mechanism modulates the composition of microorganisms on the surface of the intestine and quickly identifies the infiltrating bacteria to suppress inflammatory responses.^31^, ^32^, ^33^

Minimizing the contact of microbes in the lumen with the surface of intestinal epithelial cells is key for maintaining homeostasis. This process requires mucus, antimicrobial peptides and IgA to strengthen the physical barrier. The mucous glycoproteins secreted by the goblet cells of the intestinal epithelium form a mucus layer that coats the surface of the epithelial cells to restrain bacterial penetration and direct contact of bacteria with epithelial cells.^34^, ^35^ It is known that intestinal epithelial cells, goblet cells and Paneth cells can produce antibacterial peptides, which can kill bacteria directly by certain enzymes that attack their cell wall or disrupt their inner membrane.^36^ Gut microbiota produce IgA by regulating non‐T cell‐independent and T cell‐dependent pathways while converting B cells into IgA^+^ plasma cells in the lamina propria of the intestinal mucosa.^37^ IgA can pass through the epithelial cell layers and be secreted from the surface of epithelial cells, causing the adhesion and wrapping of harmful pathogens, and reduced bacterial movement by binding to the bacterial flagellin, thereby preventing bacteria from infiltrating into the host tissues.^4^, ^38^, ^39^ Through the above pathways, most of the bacteria can be blocked outside of the epithelial cell barriers.

Gut microbiota usually protect the host from pathogens and maintain tolerance to commensals while producing certain immune effectors for maintaining equilibrium and avoiding harmful overreactions.^40^, ^41^ Moreover, gut microbiota can selectively reduce the exposure of the resident commensal microorganisms to the surface of the intestine, regulating homeostasis in the body via group 3 innate lymphoid cells (ILC3).^42^ ILC3 shares a common developmental origin with T cells, protects against infection and produces interleukin‐22 (IL‐22). As an essential cytokine, IL‐22 promotes the healing of epithelial cells during homeostasis and infection and induces the production of antimicrobial peptides.^40^, ^41^, ^42^, ^43^ It has been shown that PGE_2_ promotes ILC3 proliferation and IL‐22 production in vivo via the PGE_2_‐EP4 signalling pathway, thereby strengthening intestinal immune function and inhibiting inflammatory responses.^44^ Several bacteria (i.e., *Bacteroides*) that are symbiotic with the host can ingest the carbohydrates produced by the host in a unique foraging way; that is, when the symbiotic microorganisms properly stimulate ILC3, IL‐22 produced by ILC3 can stimulate the fucosylation of intestinal epithelial cells. The terminal fucose fragment can be catalyzed by bacterial‐derived fucosyltransferase 2 (Fut2), which is not conducive to bacterial colonization.^45^ Interestingly, recent studies have found that IL‐22 in the intestine can increase the abundance of *Koalabacteria* by promoting glycosylation of the host's mucus *N‐*acetylglucosamine (GlcNAc), and this enriched microbe subsequently inhibits the growth of *Clostridium difficile* by consuming succinate.^46^ The regulation of the intestinal ecosystem leads to the production of bactericidal lectins via intestinal epithelium‐regenerated islet‐derived protein 3 (RegIII), including RegIIIβ and RegIIIγ.^47^ ILC3 is necessary for the expression of RegIIIγ in epithelial tissues to inhibit bacterial invasion of the intestinal epithelium.^48^ In the above two ways, the symbiotic microorganisms could play an important role in maintaining the gut microbiota composition and homeostasis.

The immune system can identify and eliminate bacteria that penetrate intestinal epithelial cells. This mucosal immune mechanism involves the uptake and phagocytosis of innate immune cells and T cell‐mediated responses. In innate immunity, mononuclear phagocytes such as macrophages (MΦ) and dendritic cells (DCs) serve as sentinels for peripheral tissues and are present in the lamina propria.^49^ Lamina propria macrophages can rapidly engulf the invading bacteria and kill the ingested pathogens by producing antibacterial peptides and reactive oxygen species. Upon damage to the epithelial cell barrier, the macrophages would be recruited around the damaged area, secreting growth factors to promote epithelial cell proliferation.^50^, ^51^ DCs are present in the lymphoid follicles and lamina propria near the intestinal epithelial cell layer, through pattern recognition receptors (PRRs) such as toll‐like receptors (TLRs) on the membrane surface to recognize components of the invading microbe and thereby activate themselves.^52^, ^53^ Interleukin 12 (IL‐12) secreted by DCs can promote the differentiation of T‐helper 1 (Th1) subsets and the expression of a proliferation‐inducing ligand (APRIL) and B cell activation factor (BAFF) to stimulate the production of IgA^+^ plasma cells.^54^, ^55^ DCs can enhance specific and humoral immunities, while stimulating the maturation of intestinal mucosa‐associated lymphoid tissues. In comparison with macrophages, DCs display weak biocidal activity, allowing the encased live bacteria to survive for a long time and be transported to mesenteric lymph nodes (MLNs). Moreover, MLNs act as a ‘firewall’ to separate the mucosal immune system from the systemic immune system, preventing antigenic components from entering lymph nodes through a series of immune responses and restraining live intestinal bacteria from penetrating the whole body.^39^ In addition, DCs have the ability to generate primary T‐cells response, whereas MΦ conduce to secondary T‐cells response.^56^ Antigen‐stimulated initial CD4^+^ T cells can proliferate and differentiate into functionally distinct subsets of T cells, such as Th1, Th2, Th17 and Treg cells^57^; among them, Treg cells suppress inflammation and maintain immune tolerance, whereas Th17 cells mediate the inflammatory response, restrain bacterial infection and strengthen the intestinal mucosal barrier. Clearly, both Treg cells and Th17 cells interact with each other and play a leading role in maintaining the homeostasis of intestinal immunity.^4^ Another study has revealed that compared with sterile mice (germ‐free, GF), mice with certain symbiotic bacteria such as filamentous fungi (segmental filamentous bacteria [SFB]) exhibited host‐specific adhesion to small intestinal epithelial cells, accompanied by specific induction of Th17 cells. In addition, SFB can induce CX3CR1^+^ monocytes to produce IL‐23 and activate ILC3 to secrete IL‐22, producing epithelial serum amyloid A (SAA) in a STAT3‐dependent manner and promoting local effector Th17 responses.^58^, ^59^ Th17 cells have a role in the development of autoimmune disease by producing the pro‐inflammatory cytokines interleukin‐17A (IL‐17A) and IL‐17F, which promote neutrophil recruitment and activation, and IL‐22, which inhibits T cell production.^41^ Treg cells are important for gut tolerance. Although symbiotic bacteria are not necessary for the production of Treg cells, they still affect their generation and function. Several studies have shown that commensal bacteria do not affect the proportion of Treg cells; however, they increase the frequency of Treg production.^57^ Extensive research on various symbiotic microorganisms, including *Lactobacillus*, *Bacteroides* and *Clostridia*, revealed that *Clostridia* clusters XIVa, IV and XVIII are correlated with an elevated frequency of Treg cells in the colon and induce the production of important anti‐inflammatory molecules, such as interleukin‐10 (IL‐10) and inducible T‐cell co‐stimulator (ICOS).^60^ In fact, IL‐10 not only can inhibit TH17 and TH1 cells in turn but can induce Vav1 in macrophages, thereby activating Rac1 to promote apoptotic cell internalization.^61^ Moreover, *Clostridia*‐produced short‐chain fatty acids (SCFAs) can not only activate the signalling pathway via GPR109a to induce anti‐inflammatory responses in DCs but also promote Treg cell proliferation and differentiation by activating GPR43 or inhibiting histone deacetylase.^62^, ^63^, ^64^ In addition, the SCFAs can regulate mucus production, IgA secretion and the expression of antimicrobial peptides, strengthening the physical barriers and maintaining the mucosal immune balance.^65^, ^66^, ^67^

### 2.4 Gut–lung axis (GLA)

**FIGURE 1:**
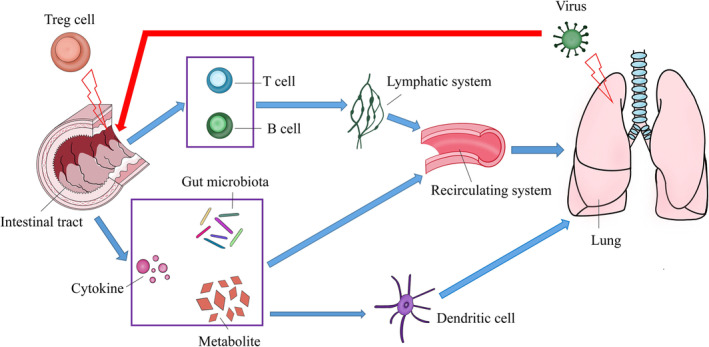
The main pathway of influence within the gut–lung axis.

The gut and lungs are anatomically distinct, but potential anatomic communications and complex pathways involving their respective microbiota have emphasized the existence of a GLA.^68^, ^69^ The gut microbiota has a long‐term effect on the mucosal immune system. Recently, Ipci et al.^70^ proposed a theory of ‘common mucosal response’ that highlights a link between the immunomodulatory activity of gut microbiota and changes in the immune function of the respiratory tract. In this case, antigen presentation‐induced lymphocytes could migrate from one certain mucosal site to other mucosal sites, including distant lung mucosa. The migration of lymphocytes may be related to the homing of T and B cells, and those lymphocytes can be home to normal mucocutaneous tissues and non‐lymphoid tissues in the inflammatory state. He et al.^38^ have suggested that naive T and B cells are initially activated in the intestine and then migrate to the mesenteric lymph nodes (MLNs) and thoracic duct to enter into the bloodstream. The distribution of T and B cells in different organs, including the lungs, can affect lung immunity. In fact, gut microbiota may trigger the homing of T and B cells by interacting with DCs and expressing the intestinal homing markers CCR9 and α4β7 imprinted on lymphocytes, promoting the immune response of the lungs.^54^, ^71^, ^72^ Bacteria and their fragments, endotoxins, cytokines or metabolites in the intestine can be transported to the systemic circulation via the intestinal barrier to regulate the immune responses.^68^, ^73^ For example, SFB, one type of indigenous microbe in the small intestine, plays an important role in mucosal immunity and in the early onset of arthritis. SFB‐induced intestinal Th17 cells are preferentially recruited to the lung to elicit lung pathological reactions.^74^ Meanwhile, mucosal DCs may be important in the GLA. It has been shown that neonates who develop atopy or asthma in childhood exhibit elevated concentrations of faecal 12,13‐diHOME, which may hinder immune tolerance. 12,13‐diHOME may cause an alteration in the in vitro expression of PPARγ‐regulated genes in DCs, reducing the production of anti‐inflammatory cytokines such as IL‐10 and subsequently decreasing the number of T cells in the lungs.^75^ There are a limited number of studies showing the detection of SCFAs in the lungs probably due to the fact that the gut microbiota is insufficient to directly affect the respiratory tract. However, intestine‐derived SCFAs may stimulate the generation of DC precursors in the bone marrow, whereas phagocytic DCs colonize the lungs to promote the differentiation of CD4^+^ T cells into Treg cells and impair Th2 differentiation, creating an anti‐inflammatory environment.^76^ The above observations provide evidence that gut metabolites have an impact on the lungs. Remarkably, patients infected with the respiratory influenza virus often have gastrointestinal‐like symptoms once the lung injury appears. In this case, the viral infection alters gut microbiota composition to promote the production of Th17 cells, triggering intestinal immune injury.^77^ Indeed, disruption of the GLA has led to increased susceptibility to airway diseases such as chronic obstructive pulmonary disease (COPD). Patients suffering from chronic gastrointestinal diseases such as irritable bowel syndrome (IBS) and inflammatory bowel disease (IBD) have higher rates of lung diseases than normal subjects.^78^, ^79^ All these findings indicate that the interaction between the gut and lung is bidirectional, and the GLA is very crucial for maintaining homeostasis (Figure 1).

## 3 RESULTS

### 3.1 The relationship between asthma and bronchiolitis in children

**FIGURE 2:**
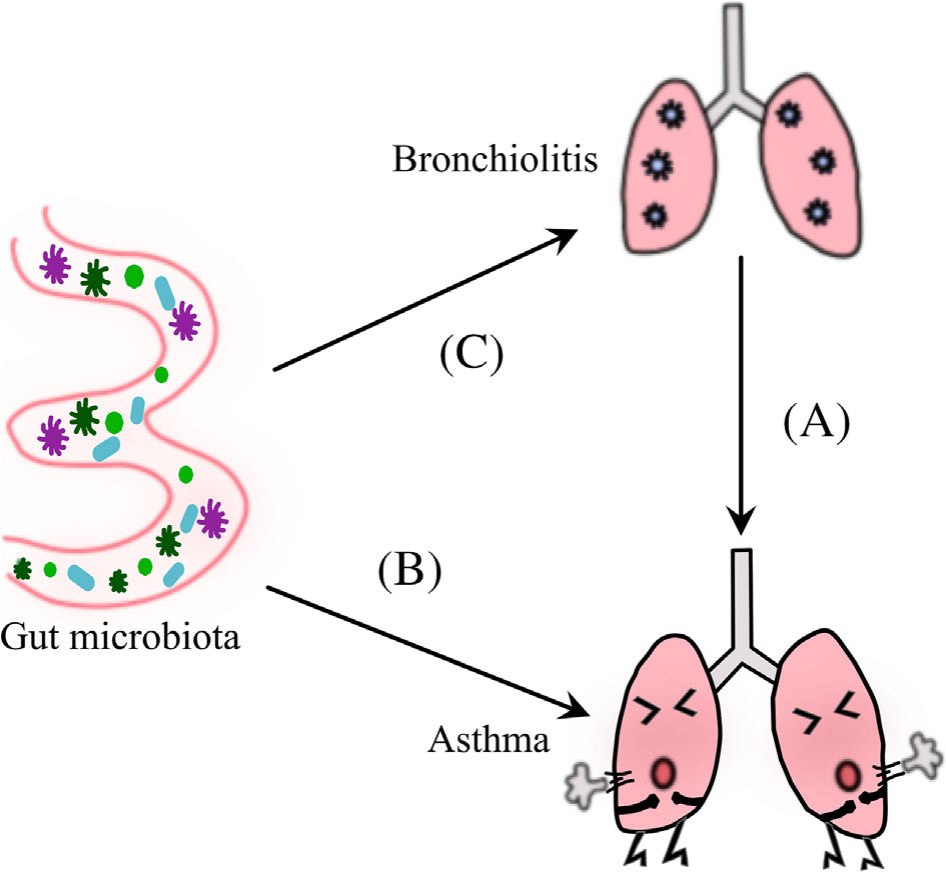
(A) Infants suffer from bronchiolitis with high IFN‐α and TNF‐γ response or Treg programming is defective in fancy may induce asthma. (B) The switch from Th1/Th2 balance to Th2 dominance in patients with gut microbiota dysbiosis may increase the chance of suffering from asthma. Asthma‐related IL‐4R‐encoding gene polymorphisms can promote the switching of iTreg cells to Th17 cell‐like cells. iTreg cell deficiency caused spontaneously developed pronounced Th2 type pathologies in the lung with hallmarks of allergic inflammation and asthma. (C) Acetic acid produced by intestinal microorganisms can induce the production of IFN‐β in the lungs, whereas promoting type 1 IFN responses under the activations of G protein‐coupled receptor 43 (Gpr43) and transcription factor NF‐κB. IFN‐1 receptor (IFNAR) regulates the resistance of acetic acid to respiratory syncytial virus (RSV) infection.

Bronchiolitis and asthma are both airway inflammatory diseases. Bronchiolitis is a common lower respiratory tract infection in infants that is mainly caused by viruses of bronchotropic epithelial cells, of which respiratory syncytial virus (RSV) is the most common pathogen and also the most likely to cause severe disease and even outbreaks.^80^ High morbidity and mortality occur in infants under 2 years old due to severe RSV infection. Some prospective studies abroad have confirmed that children with bronchiolitis are at increased risk of recurrent wheezing and asthma in the future.^81^, ^82^, ^83^ And primary research confirmed that RSV bronchiolitis is associated strongly with asthma during the first decade of life, whereas recent findings suggest that rhinovirus‐induced bronchiolitis is a stronger risk factor that may cause asthma.^84^, ^85^ Recent studies have proved that once infants suffer from bronchiolitis with a high IFN‐α and TNF‐γ response, which also increases a significantly higher risk of developing childhood asthma^86^ (Figure 2A). In addition, a recent study shows that once Treg programming is defective in infancy, bronchiolitis may induce subsequent asthma.^87^ It was a surprise that by integrating the data from a multicenter prospective cohort study of 140 infants with severe bronchiolitis, Tadao et al.^88^ identified three clinically and biologically distinct endotypes that can cause bronchiolitis.

### 3.2 Relationship between gut microbiota and bronchial asthma

Bronchial asthma is the most common chronic respiratory disease in childhood, which is characterized by airway inflammation and hyperresponsiveness. ‘Hygienic hypothesis’‐based ‘microbial hypothesis’ clarifies the cause of asthma.^89^ According to this hypothesis, the gut microbiota is closely related to the immunological mechanisms. In this case, the excessive use of antibiotics and changes in diet or lifestyle can lead to gut microbiota dysbiosis linked to immunological tolerance and hypersensitivity in humans.^89^, ^90^ A study on neonatal gut microbiota found that the low abundance of bacteria such as *Bifidobacterium* and *Akkermansia* in the gut increases the risk of suffering from asthma in the future. This observation can be attributed to the dysfunction of the neonatal gut microbiota that may cause an alteration in the proportion and function of CD4^+^ T cells.^91^ It is widely believed that asthma is related to Th1/Th2 imbalance in terms of pathogenesis. The switch from Th1/Th2 balance to Th2 dominance in patients with gut microbiota dysbiosis may increase the chance of suffering from asthma.^92^ However, recent in‐depth studies on gut microbiota revealed that the immune imbalance of Th17/Treg cells can be involved in the occurrence of asthma. Bronchoalveolar lavage fluid (BALF) test in asthma patients showed that significant presence of Th2/Th17 dual‐positive cells in the lungs was positively correlated with severe symptoms.^93^ In fact, asthma‐related IL‐4R‐encoding gene polymorphisms can promote the switching of iTreg cells to Th17 cell‐like cells, inducing the production of inflammatory factors and chemokines to modulate immune responses.^94^ Josefowicz et al.^95^ reported that a highly selective blockage in the differentiation of iTreg cells did not lead to increased pro‐inflammatory Th1 and Th17 cell responses. However, iTreg cell deficiency caused spontaneously developed pronounced Th2 type pathologies in the gastrointestinal tract and lungs with hallmarks of allergic inflammation and asthma. Collectively, it appears that both Th1/Th2 and Th17/Treg imbalances are implicated in the pathogenesis of asthma (Figure 2B).

### 3.3 Relationship between gut microbiota and bronchiolitis

Bronchiolitis is a common lower respiratory tract infection in infants and young children. Infection with a virus, especially the RSV, is the common cause of bronchiolitis, accounting for more than 40% of the cases.^96^ High morbidity and mortality occur in infants under 2 years old due to severe RSV infection. Infancy is the critical period for gut microbiota development. The gut microbiota via certain pathways may act in bronchiolitis. The pathogenesis of bronchiolitis is much more complicated and goes beyond the Th1/Th2 imbalance mechanism. It has been reported that acetic acid produced by intestinal microorganisms can induce the production of IFN‐β in the lungs while promoting type 1 IFN responses under the activation of G protein‐coupled receptor 43 (Gpr43) and transcription factor NF‐κB. IFN‐1 receptor (IFNAR) regulates the resistance of acetic acid to RSV infection, thereby alleviating the symptoms of pneumonia^97^ (Figure 2C). Recently, a study found that probiotics could exert an antiviral response via alveolar macrophage‐derived IFN‐β and suppress RSV infection to protect against pneumonia.^98^ Interestingly, Groves et al.^99^ found that the gut microbial composition and abundance increased after RSV infection in the lungs. However, the relationship between the mechanism of bronchiolitis and gut microbiota needs to be determined.

## 4 CONCLUSIONS

By retrospecting the relevant studies, this paper reviews the impact of gut microbiota on airway inflammation‐induced wheezing in children, in which the GLA plays a key role. Gut microbiota dysbiosis not only regulates the immune response of the gastrointestinal tract but also affects the immunity of distal organs such as the lung and respiratory tract,^100^ which may increase the risk of wheezing illness caused by airway inflammation in children. In recent years, numerous studies have found that appropriate probiotics,^101^, ^102^ oral vaccines^103^ or faecal bacteria transplants^104^ can be used to prevent lung infections by regulating gut microbiota, highlighting the importance of gut microbiota composition in controlling respiratory diseases.^105^ Optimizing gut microbiota, therefore, is an effective and beneficial regimen for the clinical treatment of respiratory diseases. So far, studies on other intestinal pathogens such as viruses, mycoplasmas and fungi are still lacking, and the mechanism of the GLA remains to be elucidated. Investigation of the effects of these gut pathogens on the lungs could provide a practical and reliable basis for the clinical treatment of respiratory diseases.

## AUTHOR CONTRIBUTIONS

Sichen Xue and Manhuan Xu drafted the manuscript, and Manhuan Xu and Rukkaiya Abdullahi collected the data. Sichen Xue, Rukkaiya Abdullahi and Miaoshang Su provided necessary logistic support and formal analysis. Sichen Xue, Miaoshang Su, Naisheng Wu and Jishan Zheng provided critical comments on the manuscript. All authors have read and approved the manuscript.

## CONFLICT OF INTEREST STATEMENT

The authors declare that they have no conflict of interest.

## ETHICS STATEMENT

Not applicable.

## Data Availability

The datasets used during the current study are available from the corresponding author upon reasonable request.
